# Regulatory B cells in autoimmune rheumatic diseases

**DOI:** 10.31138/mjr.28.2.75

**Published:** 2017-06-27

**Authors:** Lazaros I. Sakkas

**Affiliations:** Department of Rheumatology and Clinical Immunology, Faculty of Medicine, School of Health Sciences, University of Thessaly, Larissa, Greece

**Keywords:** Breg cells, rheumatoid arthritis, systemic lupus erythematosus, systemic sclerosis, vasculitis, dermatomyositis

## Abstract

**Background:**

Regulatory B cells (regulatory B cells, Breg cells) in recent years have been shown to be important immunoregulatory factors.

**Aim:**

To review the role of Breg cells in autoimmune rheumatic diseases.

**Methods:**

This descriptional review was carried out after research on PubMed using the keywords “Bregs and rheumatoid arthritis”, “systemic lupus erythematosus”, “Sjögren’s syndrome”, “systemic sclerosis”, “vasculitis”, and “dermatomyositis”.

**Results:**

Breg cells have an inhibitory effect on pro-inflammatory Th1 and Th17 cells and prevent the development of autoimmune diseases. Breg cells mediate their effects through interleukin-10 (IL-10, IL-10+Breg cells), but recently other Breg cells have been recognized that mediate their effects through IL-35 (IL-35+Breg cells), or through transforming growth factor-β (TGFβ, TGFβ+Breg cells). In experimental models of autoimmune diseases, Breg cells are decreased, and when expanded ex vivo and re-infused back into animals, they ameliorate disease. In humans, IL-10+Breg cells are decreased in active autoimmune diseases, such as rheumatoid arthritis, ANCA-associated vasculitis, and systemic sclerosis, and may increase to normal levels in disease remission.

**Conclusions:**

The deficiency of IL-10+Breg cells during active autoimmune rheumatic disease suggests that Breg cells may be used as biomarkers and be a possible therapeutic target in these diseases.

## INTRODUCTION

The adaptive immune system, in order to restrict the immune response to a pathogenic agent and prevent autoimmunity is equipped with regulatory cells, the regulatory T cells (Treg cells). During the last 15 years it has been shown that a subset of B cells also exhibits immunoregulatory functions, the regulatory B cells (Breg cells) This study is a descriptive review. The author searched the PubMed using the keywords “Breg cells” and “rheumatoid arthritis”, “systemic lupus erythematosus”, “Sjögren’s syndrome”, “systemic sclerosis”, “dermatomyositis”, and “vasculitis”. An extensive general bibliography for Breg cells is also at the author’s disposal.

## REGULATORY B CELLS

**Figure 1:**
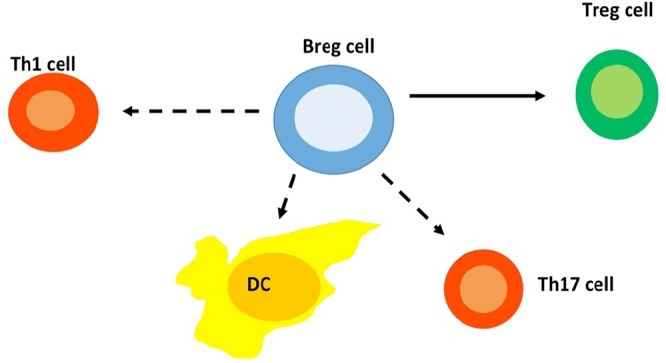
Regulatory B cells (Breg cells) inhibit pro-inflammatory Th1 and Th17 cells decrease the antigen-presenting capacity of dendritic cells (DCs) and sustain/expand regulatory T cells (Treg cells).

Breg cells, although a small proportion of peripheral blood B cells, play a major role in controlling the immune response and preventing autoimmunity. Breg cells that have been studied most thoroughly are those that produce interleukin-10 (IL-10, IL-10+Breg cells, B10 cells). B10 cells, through IL-10 production, inhibit Th1 and Th17 cells and sustain/enhance Tregs and ameliorate experimental arthritis. ^1^ B10 cells also decrease activation of macrophages and dendritic cells and their tumor necrosis factor-α (TNFα) production ^2,3^ and the antigen-presenting capacity of dendritic cells, ^4^ thus decreasing proliferation of T cells ( **Figure 1** ). The effect of Breg cells on T cells is mediated via IL-10, cell-to-cell contact through CTLA4, ^5^ IL-21 receptor, CD40, and MHC-class II.6 For example, B cells deficient in MHC-class II and B7 do not inhibit T cells. ^7^

However, there are other Breg cells that mediate their suppressive effect through IL-35 production (IL-35+Bregs) or through transforming growth factor-β production (TGFβ, TGFβ+Breg cells), but also through expression of other molecules, such as Foxp3 (Foxp3+CD19+CD5+Breg cells). ^8,9^ IL-35 is a heterodimer comprised of IL-12p35 and the product of Epstein-Bar virus-induced gene 3 (EΒi3, IL-12p35/EΒi3) and IL-35+Breg cells expanded with IL-35 decrease the activation and the antigen-presenting capacity of B cells. ^10^ B cell-derived TGFβ1 inhibits Th1 and Th17 immune responses through decrease of antigen-presenting capacity of dendritic cells in experimental autoimmune encephalomyelitis (EAE). ^11^

In humans, B10 cells of peripheral blood are increased in childhood (8–10 years-old) and are low in adults. ^12^ Interestingly, B10 cells are very low in advanced age where they exhibit a negative correlation with serum levels of rheumatoid factor; ^13^ a finding that is in line with the concept that deficiency of Breg cells likely contributes to loss of immunological tolerance and the development of autoimmunity. Breg cells are very low in peripheral blood and have to be increased in order to be studied. In the laboratory, classic stimuli for the detection of intracellular cytokines are phorbol 12-myristate 13-acetate (PMA) plus ionomycin which give a frequency of Breg cells <1% of peripheral blood B cells. Stimulation of B10 progenitor cells for more expansion of B10 cells requires stimulation with CpG or liposaccharide (LPS) or CD40. ^3^ In mice, B10 cells for their expansion require CD40 and IL-21receptor signaling from T cells. ^6^ A proliferation inducing ligand (APRIL) induces IL-10 production in CpG-stimulated Β cells, ^14^ whereas LPS or anti-IgM stimulation induces Foxp3 expression in B cells. ^15^

Many efforts have been made in order to identify Breg cells by cell surface markers, a requirement for functional assays. In addition, this makes their identification easy by flow cytometry. B10 cells are mainly within CD19+ CD24highCD38high (transitional Breg cells) and CD19+ CD24highCD27+ (memory Breg cells). ^3,16,17^ However, other markers have also been described for B10 cells, including CD19+CD1dhighCD5+, ^18^ and CD19+Tim-1+ (T cell immunoglobulin mucin domain-1). ^19^ One study reported that IL-10-producing Breg cells are mainly within the CD19+ CD25high population and that their regulatory effect on Treg cells was dependent on direct Breg and Treg cells contact. ^20^

## RHEUMATOID ARTHRITIS

In most studies, B10 cells are found to be decreased in rheumatoid arthritis (RA) compared to healthy controls. ^17,21–23^ CD19+CD24highCD38high Breg cells were found to be decreased in active RA relative to inactive RA and healthy controls and did not have the ability to inhibit Th17 cells and expand Treg cells. ^17^ In another study, the percentages of CD24highCD38high and CD24high-CD27+ Breg cells were similar to those of healthy controls, but B10 cells were decreased in RA and had an inverse correlation with disease activity (DAS28), serum levels of C-reactive protein (CRP) and serum levels of rheumatoid factor. ^21^ Also, CD19+CD5+CD1dhigh Breg cells were decreased in RA and correlated inversely with DAS28. ^24^ Finally, a study reported that the percentage of B10 cells did not differ from healthy controls, but exhibited an inverse correlation with DAS28. ^25^

## SYSTEMIC LUPUS ERYTHEMATOSUS

In systemic lupus erythematosus (SLE), CD19+ CD24highCD38high Breg cells were not decreased but produced less IL-10 compared to healthy controls and could not inhibit Th1 cells. ^16^ In another study, CD19+ CD24highCD27+Breg cells and IL-10+CD19+Breg cells were decreased in SLE. Interestingly, CD19+ CD24high-CD27+Breg cells had an inverse correlation with disease activity index (SLEDAI). ^26^ In SLE it seems that plasma-cytoid dendritic cells (pDCs) fail to induce the differentiation of CD24highCD38high B cells into IL-10+Breg cells. ^27^ In another study, the percentage of CD19+CD24highCD38high Breg cells did not differ from that of healthy controls, but IL-10+Breg cells were decreased in patients, particularly in those with nephritis. ^28^ Breg cells that resulted after activation of B cells with anti-IgM/IgG antibodies in SLE exhibited reduced ability to inhibit T cells. ^29^ However, other studies showed that Breg cells did not have a defect in SLE. CD5+CD1dhigh as well as CD19+CD24highCD38high Breg cells were increased and produced IL-10. ^30^ The percentage of CD25highFoxp3high Breg cells that produce IL-10 were increased in SLE and correlated with disease activity. ^31^

In SLE, plasmacytoid dendritic cells (pDCs) through interferon-α (IFNα) do not induce differentiation of B cells into Breg cells; a differentiation that occurs in healthy individuals. However, a similar defect also occurs in B cells from healthy individuals exposed to high concentrations of IFNα, suggesting that the disturbance in SLE resides in the interaction between Breg cells and pDCs. ^27^

In mice models of SLE, IL-10+Breg cells appear to have a protective role. ^16,32,33^ For example, CD5+CD1dhigh Breg cells from wild-type mice transferred to CD5+CD1dhigh Breg cells-deficient mice with SLE, significantly improved the survival of these mice. ^32^ In addition, administration of IL-35 to MRL/lpr mice improved clinical, laboratory, and pathological lupus nephritis, and lupus disease activity, and increased IL-10+Breg cells. ^34^

## SJÖGREN’S SYNDROME

In Sjögren’s syndrome, the percentage of CD19+ CD24highCD38high Breg cells was increased in active and inactive disease. ^35,36^ However, these cells were defective, since they failed to inhibit IFNγ production by T cells. ^36^

## VASCULITIDES

Breg cells were mostly studied in ANCA-associated vasculitis, and found to be decreased. ^37–39^ Percentages of IL-10+Breg cells and CD5+CD24highCD38high Breg cells were decreased in active ANCA-associated vasculitis and returned to normal in disease remission with concomitant decrease in serum ANCA levels. ^39^ In another study, CD5+CD19+ B cells were decreased in active disease, returned to normal during disease remission, and decreased before disease relapse. ^37^ These findings suggest that IL-10+Breg cells could be used as biomarkers in ANCA-associated vasculitis. However, two studies reported that in ANCA-associated vasculitis there is numerical but not functional impairment of CD19+CD24highCD27+ Breg cells. ^40,41^

In Henoch-Schönlein vasculitis, one study found that the number of IL-10+Breg cells was lower in patients with nephritis, and that IL-10+Breg cells correlated inversely with 24-hour urine protein. ^42^

In giant cell arteritis and polymyalgia rheumatica, one study showed that percentages of IL-10+Breg cells were within normal levels. ^43^

## DERMATOMYOSITIS

In dermatomyositis there was a decrease in CD19+ CD24highCD38high Breg cells, particularly in patients with interstitial lung disease and in patients with disease-specific autoantibodies. ^44^

## SYSTEMIC SCLEROSIS

Many lines of evidence suggest that T cells and B cells are implicated in the pathogenesis of systemic sclerosis (SSc). ^45,46^ Recently, two studies reported that Breg cells are decreased in systemic sclerosis (SSc). CD19+ CD24highCD38high transitional Bregs and CD19+ CD24highCD27+ memory Breg cells were decreased and impaired in their production of IL-10, particularly in SSc-associated interstitial lung disease. ^47^ There was a decrease in STAT3 and p38MAPK signaling in B cells. ^47^ Interestingly, Breg cells showed an inverse correlation with disease-specific autoantibodies, anti-topoisomerase I and anticentromere antibodies. ^48^

## CONCLUSIONS AND FUTURE PERSPECTIVES

In recent years, Breg cells have been found to represent significant immunoregulatory cells that suppress inflammatory immune responses and prevent autoimmunity. In autoimmune rheumatic diseases, IL-10+Breg cells are decreased and may return to normal during remission. In addition, they may decrease prior to disease flare. Therefore, Breg cells may be used as a biomarker, and be an attractive therapeutic target in these diseases. For instance, ex vivo expansion of Β10 cells with CD40 and IL-21 receptor signaling and re-infusion into mice with established EAE ameliorates disease symptoms. ^6^ However, in EAE, Β10 cells suppress disease onset whereas Treg cells suppress long-standing disease. ^4^ In humans, B10 cells may be resistant to killing by anti-CD20 monoclonal antibody (rituximab). ^2^

Cells that may give large percentages of B10 cells with IL-35 and IL-21 stimulation include bone marrow, umbilical cord blood, and fat-derived mesenchymal stem cells. Bone marrow cells, stimulated with toll-like receptor 9 (TLR9) result in large percentages of CpGproB cells that differentiate into B10 cells and inhibit ΕΑΕ in mice. ^49^ In umbilical cord blood, there are increased percentages of Β10 cells. ^5^ In addition, fat-derived mesenchymal stem cells expand B10 cells and could be used therapeutically, as has been successfully applied to a mouse model of SLE. ^50^
